# Co-Occurring Potentially Actionable Oncogenic Drivers in Non-Small Cell Lung Cancer

**DOI:** 10.3389/fonc.2021.665484

**Published:** 2021-06-16

**Authors:** Yiming Zhao, Shuyuan Wang, Zhengyu Yang, Yu Dong, Yanan Wang, Lele Zhang, Hai Hu, Baohui Han

**Affiliations:** ^1^ Department of Medical Oncology, Sun Yat-sen Memorial Hospital, Sun Yat-sen University, Guangzhou, China; ^2^ Department of Pulmonary Medicine, Shanghai Chest Hospital, Shanghai Jiao Tong University, Shanghai, China

**Keywords:** non-small cell lung cancer, actionable oncogenic drivers, *EGFR*, tyrosine kinase inhibitors, next-generation sequencing

## Abstract

**Background:**

Several oncogenic drivers in non-small cell lung cancer (NSCLC) are considered actionable with available or promising targeted therapies. Although targetable drivers rarely overlap with each other, there were a minority of patients harboring co-occurring actionable oncogenic targets, whose clinical characteristics and prognosis are not yet clear.

**Methods:**

A total of 3,077 patients with NSCLC who underwent molecular analysis by NGS were included, and their demographic and clinical data were retrospectively collected.

**Results:**

Our study found that the frequency of NSCLC patients harboring co-occurring potentially actionable alterations was approximately 1.5% (46/3077); after excluding patients with *EGFR*-undetermined mutations, the incidence was 1.3% (40/3077); 80% (37/46) harbored both *EGFR* mutations and other potentially actionable drivers such as *MET* amplification (21.6%; 8/37) and alterations in *ERBB2* including mutations (27%; 10/37) and amplification (21.6%; 8/37); other combinations of potentially actionable drivers including alterations in *ERBB2*, *KRAS*, *MET*, *ALK*, and *RET* were also identified. Additionally, *de novo MET*/*ERBB2* amplification in patients harboring *EGFR*-mutant NSCLC treated with first-generation *EGFR* tyrosine kinase inhibitors (TKIs) was associated with shorter PFS (p < 0.05). The efficacy of TKIs in NSCLC patients harboring other co-occurring potentially actionable drivers varied across different molecular subtypes.

**Conclusions:**

Approximately 1.5% of NSCLCs harbored co-occurring potentially actionable oncogenic drivers, commonly involving *EGFR* mutations. Co-occurring actionable targets may impact the efficacy of TKIs; therefore, future clinical trials in these patients should be anticipated to tailor the combination or sequential treatment strategies.

## Introduction

The heterogeneity of non-small cell lung cancer (NSCLC) is mainly determined by different oncogenic drivers (1). Although dozens of oncogenic drivers are considered to be involved in the development of lung cancer, there are only several actionable targets with widely available targeted therapies, such as *EGFR* mutations, *ALK* rearrangements, *ROS1* rearrangements, *BRAF* V600E mutation, *NTRK* rearrangements, and *RET* rearrangements (2–4). The targeted therapies for *MET* alterations (exon 14 splicing site mutations also known as skipping mutations or amplification), *ERBB2* alterations (mutations or amplification), and *KRAS* G12C mutation also demonstrated promising efficacies in clinical trials, paving a way for precision medicine of NSCLC (4–8).

More and more targeted drugs were put into the first-line setting, greatly influencing the treatment strategies; however, even with the same type of actionable drivers, the efficacy of targeted therapies varies from patient to patient (9). Several studies have proved that both progression-free survival (PFS) and overall survival (OS) of *EGFR* mutant or *ALK* rearranged NSCLCs with *TP53* mutations receiving *EGFR* or *ALK* TKIs, respectively, were significantly lower than those of patients without *TP53* mutations (10–12). Later, increasing evidence has demonstrated that other concomitant alterations such as *RB1* mutations or *PIK3CA* amplification also accelerated the resistance to *EGFR* TKIs (13, 14). In addition to these common co-existing mutations without available targeted drugs, co-occurring targetable oncogenic drivers can also be found in a small number of NSCLCs (15–18); however, there is still little evidence to make precision treatment plans for these patients, whose demographic and clinical characteristics remained largely unknown.

Based on a large population who underwent next-generation sequencing (NGS) in Shanghai Chest Hospital, our study revealed the characteristics and prognosis of NSCLC patients with co-occurring potentially actionable oncogenic drivers, trying to optimize the treatment strategies.

## Patients and Methods

### Patients

Between March 2018 and June 2019, patients with NSCLC analyzed for possible actionable targets by NGS in Shanghai Chest Hospital were enrolled. All patients were diagnosed as adenocarcinoma, squamous cell carcinoma, and other NSCLCs according to World Health Organization criteria assessed by experienced pathologists. The baseline clinical and demographic characteristics including age, gender, pathology, and stage were retrospectively collected. Our study has been approved by the institutional review board of Shanghai Chest Hospital. Written consent forms were obtained from patients before all invasive procedures and initiation of tyrosine kinase inhibitors (TKIs).

### Next-Generation Sequencing

NGS is routinely carried out for patients with advanced NSCLCs, especially adenocarcinomas, in our center unless they refuse to do so. Patients with early stage NSCLCs can also choose to receive NGS in case of recurrence. A total of 3,077 formalin-fixed, paraffin-embedded (FFPE) tumor samples acquired from resected lung or small biopsies from NSCLCs were prepared according to standard procedure. Samples with more than 5% tumor content were sent for NGS. Tissue DNA was extracted by QIAamp DNA FFPE Tissue Kit (Qiagen, Hilden, Germany) and then evaluated with the Qubit 3.0 dsDNA assay (Life Technologies, CA, USA). DNA was fragmented by the Covaris M220 Focused-Ultrasonicator (Covaris, Woburn, MA), followed by end repair, phosphorylation, and adaptor ligation. Fragments of 200–400 bp in length were selected using Agencourt AMPure beads (Beckman Coulter, Fullerton, CA, USA), followed by hybridization with capture probes baits, hybrid selection with magnetic beads, and PCR amplification. After evaluating the quality and size of the fragments by a high-sensitivity DNA assay, the samples were sequenced on a Nextseq500 sequencer (Illumina, Inc., San Diego, CA, USA) with paired-end reads. A panel of 68 cancer-related genes described previously (19, 20) were used to detect the genetic alterations of our patients, and the details of our panel are also listed in **Supplemental Table 1**. The mean depth of was >1,000×. The sequencing data in the FASTQ format were mapped to the human genome (hg19) using BWA aligner 0.7.10. Local alignment optimization, variant calling, and annotation were assessed using GATK 3.2, MuTect, and VarScan, respectively. DNA translocation analysis was performed using both Tophat2 and Factera 1.4.3. Gene-level copy number variation was assessed using a *t* test statistic after normalizing read depths at each region by total read number and region size, and correcting GC bias using a locally estimated scatterplot smoothing (LOESS) model.

### Defining MET/ERBB2 Amplification According to NGS

Although no consensus exists on the cut-off gene copy number (GCN) for *MET* amplification detected by NGS; however, GCN ≥4 as a cut-off for *MET* amplification by FISH was frequently used in several clinical trials (21, 22). Moreover, a recent study from our hospital using the same sequencing technique showed that *MET*-amplified patients with GCN >4 after crizotinib treatment tended to have longer PFS compared with GCN ≤4 (23). Therefore, the GCN for *MET* amplification in our study was greater than 4.

Similarly, there is still no recommended cut-off for *ERBB2* amplification in NSCLC. However, *ERBB2* amplification using *in situ* hybridization (ISH) in breast cancer according to 2018 ASCO/CAP clinical practice guideline was defined as *ERBB2* GCN ≥6 (24). As a result, an *ERBB2* GCN ≥6 was considered amplified in this study.

### Treatment and Follow-Up

*EGFR*-sensitizing mutations included L858R, L861Q, G719X, and S768I mutations as well as exon 19 deletions; *EGFR*-undetermined mutations refer to other mutations without well-documented clinical significance. Some of NSCLC patients harboring *EGFR*-sensitizing mutations in our study were treated with first-generation *EGFR* TKIs including gefitinib (Iressa, AstraZeneca Pharmaceuticals) (25), erlotinib (Tarceva, Roche) (26), and icotinib (Conmana, Betta) (27) at doses of 250 mg once daily, 150 mg once daily, and 125 mg three times daily, respectively, afatinib (28) (Gilotrif, Boehringer-Ingelheim), and osimertinib (29) (Tagrisso, AstraZeneca Pharmaceuticals) at doses of 40 mg once daily and 80 mg once daily, respectively. *ALK*-rearranged patients were treated with crizotinib (Xalkori, Pfizer) (30) at 250 mg twice daily or alectinib (Alecensa, Roche) (31) at 600 mg twice daily. For some patients with high-level *MET* amplification, crizotinib at 250 mg twice daily was tried. Savolitinib (Hutchison Whampoa) at 600 or 400 mg once daily was given in a clinical trial (NCT02897479) to patients with *MET* exon 14 skipping mutations. Clinical evaluation was performed every 4–6 weeks according to the Response Evaluation Criteria in Solid Tumors (RECIST1.1). Progression-free survival (PFS) was defined from the initiation of TKIs to radiographic or clinical progression or the last follow-up time (January 31, 2020).

### Statistical Analysis

The baseline characteristics between *EGFR*-mutant patients and patients with co-occurring actionable drivers receiving *EGFR* TKIs were compared with Chi-square test or two-sample t test as appropriate. Survival curves were generated for comparing PFS and OS by Kaplan–Meier methods and further compared by the log-rank test. A P value of  <0.05 was considered statistically significant. All the analyses were performed using the Statistical Package for Social Science (SPSS, Chicago, IL, USA) version 22.0 for Windows.

## Results

### Baseline Characteristics of All Patients

**Figure 1:**
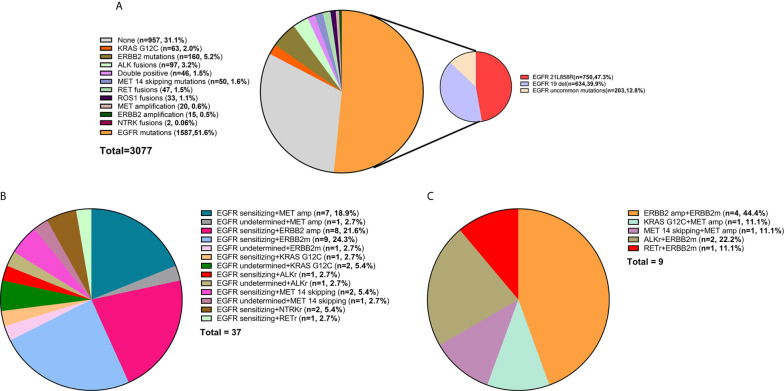
**(A)** The frequency of different oncogenic drivers in all patients. **(B)** Composition of *EGFR*-mutant NSCLC patients harboring other potentially actionable oncogenic drivers. **(C)** Composition of NSCLC patients harboring co-occurring potentially actionable drivers without *EGFR* mutations. Composition of patients with lung adenocarcinoma from MKSCC harboring co-occurring potentially actionable oncogenic drivers. double-positive, with two potentially actionable oncogenic drivers; triple-positive, with three potentially actionable oncogenic drivers; del, deletions; *EGFR* sensitizing, sensitizing *EGFR* mutations, *EGFR* undetermined, undetermined *EGFR* mutations; amp, amplification; *ERBB2*m, *ERBB2* mutation; *MET* 14 skipping, *MET* exon 14 skipping mutation; *ALK*r, *ALK* rearrangement; *NTRK*r, *NTRK* rearrangement; *RET*r, *RET* rearrangement.

A total of 3,077 NSCLC patients were analyzed for oncogenic alterations by NGS, among whom, 81% (2481/3077), 11% (333/3077), and 8% (263/3077) were diagnosed as adenocarcinoma, squamous cell carcinoma, and other subtypes, respectively. The detailed characteristics were shown in **Table 1**. Of the patients, 69% (2120/3077) harbored at least one potentially actionable oncogenic drivers, namely *EGFR* mutations, *ALK* rearrangements, *ROS1* rearrangements, *BRAF* V600E mutation, *MET* amplification, *MET* exon 14 skipping mutations, *RET* rearrangements, *NTRK* rearrangements, *ERBB2* alterations (mutations and amplification), and *KRAS* G12C mutation. Among patients with at least one potentially actionable target, 75% (1587/2120) harbored *EGFR* mutations including exon 21 L858R mutation (47%; 750/1587), exon 19 deletions (40%; 634/1587), and other uncommon mutations (13%; 203/1587). The details of all potentially actionable oncogenic targets were listed in **Figure 1A**.

**Table 1:** Demographic and clinico-pathological characteristics of all patients who underwent NGS.

Variables	
Total number of patients	3077
Age median (range)	62 (22–88)
Gender N (%)	
Male	1561 (51)
Female	1516 (49)
Smoking N (%)	
Yes	1490 (48)
No	1587 (52)
Pathology N (%)	
ADC	2481 (81)
SQCC	333 (11)
Others	263 (8)
Stage	
IA-IIIA	1915 (62)
IIIB-IV	1162 (38)
Potentially targetable drivers	
Yes	2120 (69)
No	957 (31)

ADC, adenocarcinoma; SQCC, squamous cell carcinoma.

### Baseline Characteristics of Patients Harboring Co-Occurring Potentially Actionable Targets

Of the patients, 1.5% (46/3077) had co-occurring potentially targetable oncogenic drivers. The characteristics of these patients were shown in **Table 2**. These patients are commonly seen in females (70 *vs* 30%), non-smokers (76 *vs* 24%), and adenocarcinomas (89 *vs* 11%). 80% (37/46) had *EGFR* mutations and other concomitant potentially actionable drivers, while 20% (9/46) did not harbor *EGFR* mutations. After excluding patients with *EGFR*-undetermined mutations, the remaining patients harboring co-occurring potentially actionable oncogenic drivers accounted for 1.3% (40/3077) of all NSCLCs (**Figure 1B**).

**Table 2:** The demographic and clinico-pathological characteristics of patients harboring co-occurring potentially actionable targets.

Characteristics	
**Total number of patients**	46
**Median age (range)**	62 (35–81)
**Gender N, (%)**	
Male	14 (30)
Female	32 (70)
**Smoking N, (%)**	
Non-smoker	35 (76)
Smoker	11 (24)
**Pathology N, (%)**	
ADC	41 (89)
Non-ADC	5 (11)
**Stage**	
I–IIIA	19 (41)
IIIB–IV	27 (59)

ADC, adenocarcinoma.

A total of 37 (80%; 37/46) patients harbored both *EGFR*-sensitizing (84%; 31/37) or -undetermined (16%; 6/37) mutations and other oncogenic drivers. The concomitant potentially targetable drivers included *de novo MET* amplification (21.6%; 8/37), *de novo ERBB2* amplification (21.6%; 8/37), *ERBB2* mutations (27.0%; 10/37), *KRAS* G12C mutation (8.1%; 3/37), *ALK* rearrangements (5.4%; 2/37), *MET* exon 14 skipping mutations (8.1%; 3/37), *NTRK* rearrangements (5.4%; 2/37), and *RET* rearrangements (2.7%; 1/37). All the molecular subtypes of these patients were shown in **Figure 1B**.

Among the patients harboring co-occurring potentially actionable targets without *EGFR* mutations, 44.4% (4/9) had both *ERBB2* amplification and *ERBB2* mutations; 11.1% (1/9) had *KRAS* G12C mutation and concurrent *MET* amplification; 11.1% (1/9) had *MET* amplification and a concurrent *MET* exon 14 splicing mutation; 22.2% (2/9) harbored both *ALK* rearrangements and *ERBB2* mutations, and one patient (11.1%; 1/9) had *RET* rearrangement and an *ERBB2* mutation (**Figure 1C**).

### The Impact of Co-Occurring Potentially Actionable Oncogenic Drivers on Targeted Therapies

A total of 23 patients with co-occurring patterns were treated with different kinds of TKIs, whose treatment types, response, and progression events were shown in a swimmer plot (**Supplemental Figure 1**). A total of 17 *EGFR*-mutant patients with other potentially actionable oncogenic drivers were treated with first-generation *EGFR* TKIs. The demographic and clinical characteristics of these patients were listed in **Table 3** and **Supplemental Table 2**. Of patients, 29% (5/17) and 29% (5/17) harbored concurrent *de novo MET* amplifications and *ERBB2* amplifications, respectively. From March 2018 to June 2019, a total of 205 patients with *EGFR* mutations alone who underwent NGS analysis were treated with first-generation *EGFR* TKIs and had full medical records to evaluate the efficacy, whose baseline characteristics including TP53 mutation status were not significantly different from those of *EGFR*-mutant patients harboring other concurrent oncogenic drivers except that there were more females in the double-positive cohort (**Supplemental Table 3**).

**Table 3:** The characteristics of *EGFR* mutant patients harboring other potentially actionable drivers treated with first-generation *EGFR* TKIs.

Characteristics	
**Total number of patients**	17
**Median age (range)**	60 (35–72)
**Gender N, (%)**	
Male	3 (18)
Female	14 (82)
**Smoking N, (%)**	
Non-smoker	14 (82)
Smoker	3 (18)
**Pathology N, (%)**	
ADC	15 (88)
Non-ADC	2 (12)
**EGFR mutation type N, (%)**	
21L858R	12 (71)
19del	4 (23)
18G719A	1 (6)
**Concomitant alterations N, (%)**	
*MET* amplification	5 (29)
*ERBB2* amplification	5 (29)
*ERBB2* mutations	3 (18)
*MET* exon 14 splicing mutation	1 (6)
*RET* rearrangement	1 (6)
*NTRK* rearrangement	1 (6)
*KRAS* G12C mutation	1 (6)

ADC, adenocarcinoma.

**Figure 2:**
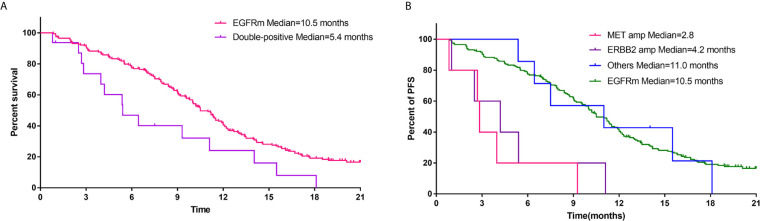
**(A)** Comparison of PFS of first-generation *EGFR* TKIs for patients with EGFR mutations alone and patients harboring both *EGFR* mutations and other potentially actionable oncogenic drivers. **(B)** PFS of first-generation *EGFR* TKIs for *EGFR*-mutant patients with concurrent *MET* amplification, *ERBB2* amplification, and other oncogenic drivers.

As shown in **Figure 2A**, *EGFR*-mutant patients with other concurrent potentially actionable drivers demonstrated significantly lower PFS compared to patients with *EGFR* mutations alone (5.4 *vs* 10.5 months; HR = 1.94, 95% CI: 1.16–3.25, p = 0.0042). Further analysis of molecular subtypes found that both *de novo MET* amplification (2.8 *vs* 10.5 months; HR = 6.03, 95% CI: 2.43–15.00; p < 0.0001) and *de novo ERBB2* amplification (4.2 *vs* 10.5 months; HR = 2.5, 95% CI, 1.03–6.09; p = 0.0005) significantly reduced PFS of *EGFR* TKIs. However, *EGFR*-mutant patients harboring other potentially targetable drivers except *MET*/*ERBB2* amplification showed no significant difference in PFS compared with patients harboring *EGFR* mutations alone (11.0 *vs* 10.5 months; HR = 1.13, 95% CI: 0.48–2.69; p = 0.76) (**Figure 2B**), although the PFS of these patients still fluctuated greatly across different molecular subtypes (**Supplemental Table 2**).

A total of six patients with co-occurring potentially targetable oncogenic drivers were treated with other TKIs. One *EGFR*-mutant patient carrying concurrent *de novo ERBB2* amplification was treated with afatinib, reaching a PFS of 4.6 months, while PFS of one *EGFR*-mutant patient with concurrent *de novo MET* amplification receiving osimertinib was 14.2 months. One *EGFR*-mutant patient with concomitant high-level *MET* amplification (copy number = 8.8) received crizotinib, yet with a PFS of only 2.5 months. One *ALK*-rearranged patient with a concurrent *ERBB2* mutation was treated with alectinib, achieving partial response. One *MET*-amplified patient with a concomitant *MET* exon 14 skipping mutation was treated with crizotinib, having satisfying response with PFS of 14.4 (**Table 4**).

**Table 4:** Characteristics of patients harboring other co-occurring actionable drivers receiving targeted therapies.

No.	Sex	Age, y	Pathology	Smoking	Therapies	Alterations	Stage	Response	PFS
1	female	42	PSC	never	Afatinib	*EGFR* exon 18 G719A + *ERBB2* amp	IV	SD	4.6
2	male	68	SQCC	current	Crizotinib	*EGFR* exon 19 del + *MET* amp (CN=8.8)	IV	SD	2.5
3	male	66	ADC	former	gefitinib+ chemo	*EGFR* exon 11 P411S + *KRAS* exon 2 G12C	IV	PR	13
4	female	41	ADC	never	Alectinib	*EML4*-ALK(E13:A20) +*ERBB2* 27A1216V	IV	PR	11.2*
5	male	70	ADC	former	Crizotinib	*MET* exon 14 skipping mutation + *MET* amp	IV	PR	14.4
6	female	61	ADC	never	Osimertinib	*EGFR* exon 21 L858R + *MET* amp	IV	PR	14.2*

*The diseases have not progressed in these patients at the time of last follow-up.

PSC, pulmonary sarcomatoid carcinoma; SQCC, squamous cell carcinoma; ADC adenocarcinoma; amp, amplification.

## Discussion

Our study found that the incidence of NSCLC patients harboring co-occurring potentially actionable alterations was approximately 1.5% (46/3077), likely to be found in females, non-smokers, and adenocarcinomas. Among these patients, 80% (37/46) harbored *EGFR*-sensitizing (84%; 31/37) or -undetermined (16%; 6/37) mutations and other concurrent potentially targetable oncogenic drivers such as *de novo MET* amplification (21.6%; 8/37) and alterations in *ERBB2* including mutations (27.0%; 10/37) and amplification (21.6%; 8/37). Other concurrent potentially actionable targets in *EGFR*-mutant patients treated with first-generation *EGFR* TKIs were associated with shorter PFS (5.4 *vs* 10.5 months; HR = 1.94, 95% CI: 1.16–3.25, p = 0.0042), suggesting that co-occurring potentially targetable oncogenic drivers may impact the efficacy of *EGFR* TKIs. Further analysis showed that it was *de novo MET*/*ERBB2* amplification that played major roles in the primary resistance to first-generation *EGFR* TKIs, while the third-generation *EGFR* TKI, osimertinib, may bring better benefits to these patients. In addition, the efficacy of TKIs in NSCLC patients harboring other co-occurring potentially actionable targets varied across different molecular subtypes with overall encouraging responses.

With the advent and rapid development of NGS, the genomic alterations of lung cancer have been fully investigated, helping to move forward to the era of precision medicine. There are three generations of TKIs for *EGFR* mutations, one of the most common oncogenic drivers in NSCLC. The first-generation *EGFR* TKIs including gefitinib, erlotinib, and icotinib were usually used as the first-line treatments in China due to their wide availability and affordable prices. Our study found that the PFS of the first-generation TKIs in *EGFR*-mutant patients was 10.5 months, similar to the results from previous clinical trials (25–27). Owing to the greatly heterogeneous responses to *EGFR* TKIs across individuals harboring *EGFR* mutations, many research studies were carried out to study the factors that may affect the efficacy, among which, concurrent genomic alterations were mostly investigated. For example, a retrospective study from Korea included patients who underwent molecular analysis by NGS before treatment with first-/second-generation *EGFR* TKIs (cohort 1) or third-generation *EGFR* TKIs after failure in the previous TKIs (cohort 2). Their results showed that *TP53* mutations were independently associated with worse outcomes in cohort 1, while in cohort 2, *TP53*, *RB1*, and *PTEN* mutations as well as *MDM2* amplifications all resulted in shorter PFS (11), suggesting that concurrent genomic alterations accelerated the resistance to *EGFR* TKIs. Additionally, co-occurring *TP53* mutations were reported to be associated with worse outcomes of *EGFR* TKIs in several studies (10, 11, 13, 32). However, these concomitant genetic alterations in previous studies were mostly considered untargetable without available drugs. There are only a few oncogenic drivers in NSCLC having commercially available or promising targeted therapies, including alterations in *EGFR*, *ALK*,*ROS1*, *BRAF*, *MET*, *RET*, *ERBB2*, *NTRK*, and *KRAS*. Although these drivers were considered mutually exclusive previously (33), increasing evidence demonstrated that a minority of NSCLCs harbored co-occurring potentially actionable alterations (17, 34–36), whose overall characteristics remained largely unknown due to the small sample size of previous reports. Our findings showed that the incidence of NSCLC patients harboring co-occurring potentially actionable alterations was approximately 1.5% (46/3077), and they were commonly found in females, non-smokers, and adenocarcinomas.

Similar results were also shown in western populations, although the frequency of oncogenic drivers, especially *EGFR* mutations, in western patients with NSCLC was reported to be lower than that of eastern population (1, 37, 38). The patients with co-occurring potentially actionable targets accounted for 3.6% (31/860) of all lung adenocarcinoma according to the data presented by MSKCC (38). Among these patients, 84% (26/31) harbored *EGFR*-sensitizing (81%; 21/26) or -undetermined (19%; 5/26) mutations with other concurrent potentially actionable drivers including *ERBB2* amplification (50%; 13/26), *MET* amplification (27%; 7/26), *KRAS* G12C mutation (11.5%; 3/26), and *ERBB2* mutations (11.5%; 3/26), similar to the eastern population. There are five (16%; 5/31) patients harboring other types of co-occurring potentially actionable targets. However, the GCNs for *MET*/*ERBB2* amplification were not known in this database.

Among NSCLC patients with co-occurring potentially oncogenic drivers, 80% (37/46) harbored *EGFR* mutations and other concurrent potentially targetable drivers, commonly involving *de novo MET* amplifications (21.6%; 8/37) and alterations in *ERBB2* including mutations (27.0%; 10/37) and amplification (21.6%; 8/37). It was found that concurrent actionable drivers, especially *MET*/*ERBB2* amplification, contributed to the primary resistance to *EGFR* TKIs. *MET* amplification often occurred in *EGFR-*mutant NSCLC after failure of previous TKIs as an acquired resistance mechanism by activating *ERBB3* signaling (39, 40). From a cytological prospective, a small proportion of *EGFR*-mutant cells already harbored *MET* amplifications before initiation of TKIs, finally resulting in drug resistance (41). A previous study showed that approximately 3.2% (5/154) of *EGFR*-mutant patients harbored concurrent *MET* amplifications before treatment with *EGFR* TKIs, and the PFS for these patients was significantly shorter than that of the patients with *EGFR* mutations alone (17), but the sample size was too small to reach a concrete conclusion. In our study, five *EGFR*-mutant patients with concurrent *de novo MET* amplifications treated with first-generation *EGFR* TKIs demonstrated worse PFS compared to the patients with *EGFR* mutations alone, further supporting the conclusion that *de novo MET* amplification contributed to the primary resistance of first-generation *EGFR* TKIs. Additionally, one *EGFR*-mutant patient with high-level *MET* amplification received crizotinib with stable disease lasting for only 2.5 months, suggesting that these patients may respond poorly to only one targeted drug. A phase Ib/II clinical trial demonstrated promising efficacy of capmatinib plus gefitinib after failure of *EGFR* TKIs in patients having *EGFR*-mutant and *MET*-amplified NSCLC with acceptable toxicities (21). Therefore, the combination regimen can also be tried in *EGFR*-mutant patients with *de novo MET* amplifications in clinical practice to overcome primary resistance. Additionally, one *EGFR*-mutant patient with both *de novo MET* amplification was treated with osimertinib, achieving prolonged partial response, suggesting the third-generation *EGFR* TKI, osimertinib, might overcome the primary resistance from *de novo MET* amplification.

Similar to concurrent *de novo MET* amplification, *de novo ERBB2* amplification was also associated with shorter PFS. Approximately 13% of patients with *EGFR*-mutant NSCLC will acquire *ERBB2* amplification after failure of first-generation *EGFR* TKIs. However, the role of *de novo ERBB2* amplification in *EGFR*-mutant NSCLC has not been fully revealed. A previous study showed that 4% (8/200) of all *EGFR*-mutant NSCLC harbored concurrent *ERBB2* amplifications before treatment with TKIs, leading to a shorter PFS compared to patients with *EGFR* mutations alone (14). In our study, it was found patients having both *EGFR*-mutant and *ERBB2* amplified NSCLC treated with first-generation EGFR TKIs reached a median PFS of 4.2 months, which was significantly shorter than that of patients with EGFR mutations alone (4.2 *vs* 10.5 months; HR = 2.5, 95% CI, 1.03–6.09; p = 0.0005). A recent phase II basket trial demonstrated a 51% response rate in 49 patients with *ERBB2*-amplified or mutant NSCLC treated with ado-trastuzumab emtansine (T-DM1), an anti-*ERBB2*/*HER2* antibody-drug conjugate (ADC), suggesting that ADCs are effective in patients with *ERBB2*-aberrant NSCLC. Therefore, the future clinical trials of *EGFR* TKIs plus ADCs can be launched in *EGFR*-mutant patients with concurrent *ERBB2* amplification.

Additionally, there were *EGFR*-mutant patients harboring other concurrent potentially actionable drivers such as *KRAS* mutations. Sotorasib, a *KRAS* inhibitor, in a recent clinical trial showed encouraging efficacy in patients with heavily pretreated advanced solid tumors harboring *KRAS* G12C mutation, paving a way for targeted therapy in *KRAS*-mutant patients. There are several gene fusions in NSCLC with corresponding targeted drugs including *ALK*, *ROS1*, *RET*, and *NTRK* (42). Although these fusions rarely overlap with other oncogenic drivers (43, 44), our study found that they can co-exist with other actionable targets such as *EGFR* mutations and *ERBB2* mutations. Moreover, there were four patients harboring both *ERBB2* mutations and *ERBB2* amplifications, although a previous study showed that *ERBB2* mutations were not associated with *ERBB2* amplification (45).

There were some drawbacks of our study. Firstly, this was a retrospective study with a relatively small sample size, which cannot avoid selection bias and reflect the entire population with co-occurring potentially actionable oncogenic drivers. Secondly, *MET* and *ERBB2* amplifications were not further confirmed by fluorescence *in situ* hybridization (FISH), although NGS was frequently applied in both clinical trials and routine practice to detect amplifications (6, 8, 21),. Furthermore, several studies showed high concordance between NGS and FISH or immunohistochemistry in detecting amplification (46–48). Additionally, the efficacy of ADCs in *ERBB2*-amplified NSCLC varied across different clinical trials (5, 6, 49); however, a recent study has revealed that the overall response rate by RECIST of T-DM1 in *ERBB2*-amplified NSCLC was 50% (5/10) (6), suggesting *ERBB2* amplification was also a promising target. Moreover, the efficacy of *EGFR* TKIs in some of the *EGFR-*undetermined mutations considered potentially actionable in our study still needed to be explored. Finally, there is a potential risk of confounders by patient clinical characteristics in the survival analysis, especially in the subgroup analysis.

## Conclusions

In conclusion, our study showed approximately 1.5% (46/3077) of all NSCLCs harbored co-occurring potentially actionable oncogenic drivers, commonly involving *EGFR* mutations; after excluding patients with *EGFR*-undetermined mutations, the incidence was 1.3% (40/3077). These patients are likely to be found in females, non-smokers, and adenocarcinomas. In *EGFR*-mutant patients, *de novo MET*/*ERBB2* amplification was associated with shorter PFS, and the combination of *EGFR* and *MET*/*ERBB2* inhibitions or third-generation *EGFR* TKIs can be tried in future to achieve better response. The efficacy of TKIs in NSCLC patients harboring other co-occurring potentially actionable drivers varied across different molecular subtypes. Many molecular subtypes of co-occurring actionable oncogenic drivers were found in our study, suggesting the complexity of oncogene-addicted NSCLC. In order to tailor the combination or sequential treatment strategies, future clinical trials for these patients should be anticipated.

## Data Availability Statement

The original contributions presented in the study are included in the article/**Supplementary Material**. Further inquiries can be directed to the corresponding authors.

## Ethics Statement

Ethical approval was not provided for this study on human participants because this was a retrospective study. The patients/participants provided their written informed consent to participate in this study. Written informed consent was obtained from the individual(s) and minor(s)’ legal guardian/next of kin for the publication of any potentially identifiable images or data included in this article.

## Author Contributions

YZ, SW, and ZY have substantial contributions to the conception or design of the work, the collection and analysis of data, the writing and editing of the article. BH and HH contributed to interpretation of data and revision of the manuscript. The rest of the authors have given substantial contributions to the work by providing editing and writing assistance. All authors contributed to the article and approved the submitted version.

## Funding

This study was supported in part by grants from the State Key Program of National Natural Science of China (8173077) and Shanghai Rising-Star Program (2YF1428100).

## Conflict of Interest

The authors declare that the research was conducted in the absence of any commercial or financial relationships that could be construed as a potential conflict of interest.

## Supplementary Material

Supplementary Figure 1 A swimmer plot for patients with co-occurring patterns treated with different kinds of TKIs. *EGFR*m, *EGFR* mutations alone; double-positive, with two potentially actionable oncogenic drivers; amp, amplification.
