# Supplementary figures and images for: Co-Occurring Potentially Actionable Oncogenic Drivers in Non-Small Cell Lung Cancer
Source: Front Oncol. 2021 Jun 16;11:665484. doi: 10.3389/fonc.2021.665484 (PMC8242190; doi:10.3389/fonc.2021.665484)

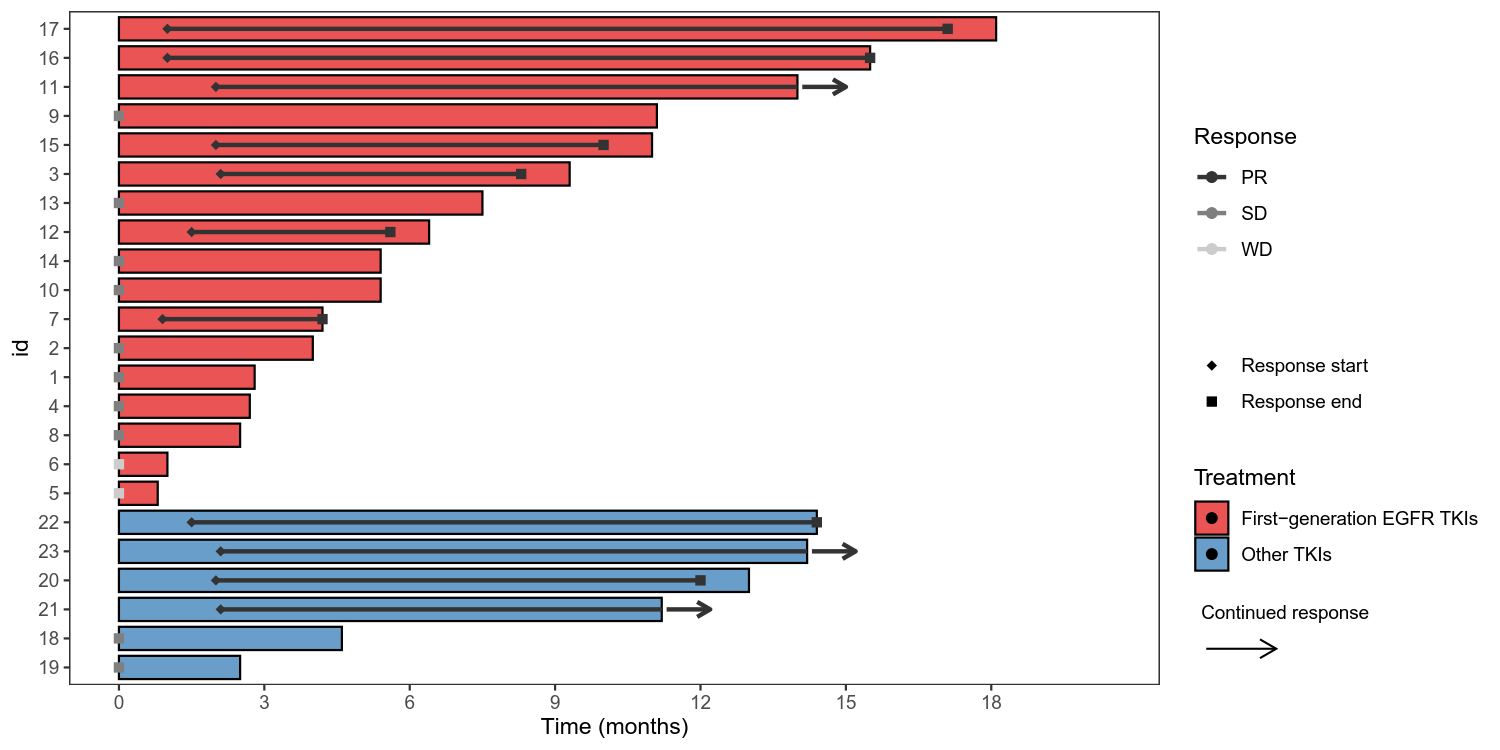

Supplement: Supplementary Figure 1 — A swimmer plot for patients with co-occurring patterns treated with different kinds of TKIs. EGFRm, EGFR mutations alone; double-positive, with two potentially actionable oncogenic drivers; amp, amplification. [file Image_1.tif]
