# Supplementary material for: Co-Occurring Potentially Actionable Oncogenic Drivers in Non-Small Cell Lung Cancer
Source: Front Oncol. 2021 Jun 16;11:665484. doi: 10.3389/fonc.2021.665484 (PMC8242190; doi:10.3389/fonc.2021.665484)
Supplement: Supplementary file 3 [file Table_2.docx]

Supplemental Table 2. List of *EGFR*-mutant patients harboring other actionable oncogenic drivers treated first-generation *EGFR* TKIs

| No. | Sex | Age, y | Pathology | Smoking | Therapies | Alterations | Stage | Response | PFS |
| --- | --- | --- | --- | --- | --- | --- | --- | --- | --- |
| 1 | female | 52 | ADC | never | icotinib | *EGFR* exon 19 del+  *MET* amp | IV | SD | 2.8 |
| 2 | female | 64 | ADC | never | icotinib | *EGFR* exon 21 L858R+  *MET* amp | IV | SD | 4.0 |
| 3 | female | 47 | ADC | never | icotinib | *EGFR* exon 21 L858R+  *MET* amp | IV | PR | 9.3 |
| 4 | male | 63 | ADC | never | icotinib | *EGFR* exon 21 L858R*+*  *MET* amp | IV | SD | 2.7 |
| 5 | female | 38 | ADC | never | icotinib | *EGFR* exon 21 L858R+  MET amp | IV | PD | 0.8 |
| 6 | male | 44 | ADC | current | gefitinib | *EGFR* exon 19 del+  *ERBB2* amp | IV | PD | 1.0 |
| 7 | male | 59 | ADC | former | icotinib | *EGFR* exon 21 L858R+  *ERBB2* amp | IV | PR | 4.2 |
| 8 | female | 50 | LCEC | never | icotinib | *EGFR* exon 21 L858R+  *ERBB2* amp | IV | SD | 2.5 |
| 9 | female | 69 | ADC | never | icotinib | *EGFR* exon 19 del+  *ERBB2* amp | IV | SD | 11.1 |
| 10 | female | 35 | ADC | never | icotinib | *EGFR* exon 21 L858R+  *ERBB2* amp | IV | SD | 5.4 |
| 11 | female | 72 | ADC | never | icotinib | *EGFR* exon 21 L858R+  *ERBB2* exon 8 S310F | IV | PR | 14.0* |
| 12 | female | 60 | ADC | never | gefitinib | *EGFR* exon 21 L858R+  *ERBB2* exon 6 P230S | IV | PR | 6.4 |
| 13 | female | 68 | ASC | former | icotinib | *EGFR* exon 21 L858R+ | IV | SD | 7.5 |
| 14 | female | 63 | ADC | never | icotinib | *EGFR* exon 18 G719A+  *PPM1L-RET(Pintergenic:R12)* | IV | SD | 5.4 |
| 15 | female | 66 | ADC | never | icotinib | *EGFR* exon 21 L858R+  *KRAS* exon 2 G12C | IV | PR | 11.0 |
| 16 | female | 56 | ADC | never | icotinib | *EGFR* exon 19 del+  *FMN2-NTRK1*(F16:N11) | IV | PR | 15.5 |
| 17 | female | 65 | ADC | never | icotinib | *EGFR* exon 21 L858R+  *MET* exon 14 skipping mutation | IV | PR | 18.1 |

***The diseases have not progressed in these patients at the time of last follow-up**

**Abbreviations:** ADC, adenocarcinoma; LCEC, large cell endocrine carcinoma; ASC, adenosquamous carcinoma.
