# Supplementary material for: Co-Occurring Potentially Actionable Oncogenic Drivers in Non-Small Cell Lung Cancer
Source: Front Oncol. 2021 Jun 16;11:665484. doi: 10.3389/fonc.2021.665484 (PMC8242190; doi:10.3389/fonc.2021.665484)
Supplement: Supplementary file 4 [file Table_3.docx]

| **Characteristics** | **Double-positive** | **EGFR mutations alone** | **p-value** |
| --- | --- | --- | --- |
| **Median Age (Range)** | 60 (35-72) | 63 (33-87) | 0.15 |
| **Gender N, (%)** |  |  | 0.04 |
| Male | 3 (17) | 89 (43) |  |
| Female | 14 (83) | 116 (57) |  |
| **Smoking N, (%)** |  |  | 0.12 |
| Non-smoker | 14 (83) | 126 (61) |  |
| Smoker | 3 (17) | 79 (39) |  |
| **Pathology N, (%)** |  |  | 1.00 |
| ADC | 15 (88) | 183 (89) |  |
| Others | 2(12) | 22 (11) |  |
| **EGFR mutation type N, (%)** |  |  | 0.22 |
| 21L858R | 12 (71) | 97 (47) |  |
| 19del | 4 (23) | 87 (43) |  |
| Uncommon | 1 (6) | 21 (10) |  |
| **Concurrent TP53 mutations N,(%)** |  |  | 0.81 |
| Yes | 11 (65) | 126 (61) |  |
| No | 6 (35) | 79 (39) |  |

Supplemental Table 3. Characteristics of NSCLC patients treated with first-generation *EGFR* TKIs harboring both *EGFR* mutations and other oncogenic drivers or *EGFR* mutations alone.

**Abbreviations:** ADC, adenocarcinoma.
